# Impact of Antihypertensive, Antihyperlipidemic, and Mixed Antidepressant Medications on Dental Implant Success: A Retrospective Cohort Study

**DOI:** 10.7759/cureus.104647

**Published:** 2026-03-04

**Authors:** Antony Chidiac, Charbel Kachouh, Richard M. Sadaka, Joseph Bassil

**Affiliations:** 1 Oral Surgery and Implantology, Saint Joseph University of Beirut, Beirut, LBN; 2 Dentistry, Faculty of Dental Medicine, Saint Joseph University of Beirut, Beirut, LBN; 3 Economics and Business Administration, Lebanese University, Beirut, LBN

**Keywords:** anticholesteremic agents, antidepressive agents, antihypertensive agents, bone remodeling, dental implants, osseointegration, survival rate

## Abstract

Objective: This study aimed to analyze the influence of antihypertensive, antihyperlipidemic, and mixed antidepressant medications on the failure and survival rates of dental implants.

Methods: Out of a total of 2,815 patients who received 5,853 dental implants between 2012 and 2022, 655 patients with 2,004 implants met the inclusion criteria and were retrospectively reviewed. Patients were divided into four groups: control group (n = 512 patients with 1,581 implants), antihypertensive group (n₁ = 74 patients with 233 implants), antihyperlipidemic group (n₂ = 40 patients with 99 implants), and mixed antidepressant group (n₃ = 29 patients with 91 implants). Implant failure was defined according to the recommendations of the Pisa Consensus Conference of the International Congress of Oral Implantologists.

Results: Kaplan-Meier analysis over a 10-year follow-up period showed significant differences in implant survival between the control group and all monotherapy groups. Implant survival was higher in patients receiving antihypertensive or antihyperlipidemic therapy and lower in those treated with mixed antidepressants. Log-rank tests confirmed statistically significant differences compared with controls (*p* < 0.05). Implant failure rates were 1.29% (n = 3/233) for the antihypertensive group, 1.01% (n = 1/99) for the antihyperlipidemic group, and 12.09% (n = 11/91) for the mixed antidepressant group, compared with 5.12% (n = 81/1581) in the control group. Mixed-effects survival analysis demonstrated a reduced risk of implant failure in patients receiving antihypertensive therapy (HR = 0.23) and antihyperlipidemic therapy (HR = 0.16), while mixed antidepressant medication was associated with a significantly increased risk of failure.

Conclusion: Mixed antidepressant medications are associated with an increased risk of dental implant failure, while antihypertensive and antihyperlipidemic agents appear to have a protective effect, contributing to improved implant survival.

## Introduction

In an era characterized by increasing life expectancy, oral health has emerged as a fundamental component of overall systemic health [1]. Dental practitioners, therefore, play a crucial role in linking oral conditions to general health status, particularly in industrialized societies experiencing improved socioeconomic standards [1]. Patients seeking implant therapy often present both aesthetic and functional demands, which strongly influence psychological well-being, social integration, and self-esteem.

The management of edentulism has evolved considerably since Per-Ingvar Branemark introduced osseointegration in 1950, laying the foundation for modern implant dentistry [2]. Today, dental implants are considered a predictable and effective treatment for partial and complete edentulism, providing durable functional and aesthetic benefits [2]. Long-term implant survival rates exceeding 90% over roughly 10 years have been consistently reported [3]. Nevertheless, implant failure remains a clinically relevant issue despite advances in surgical techniques, implant materials, and design [3].

The risk of implant failure varies among individuals and is influenced by biological, mechanical, and behavioral factors [3]. Among biological determinants, systemic medications warrant particular attention. Many implant candidates, especially older adults, are on long-term pharmacological therapy for chronic conditions [3]. Because implant success relies primarily on osseointegration, defined as direct bone-to-implant contact without interposed soft tissue, any disruption in bone metabolism may impair healing and lead to early implant loss or peri-implant complications [3]. While chronic systemic medications are known to affect bone metabolism, their long-term influence on dental implant outcomes remains insufficiently explored [4]. Hypertension, hyperlipidemia, and depression are highly prevalent chronic conditions.

Antihypertensive medications, commonly prescribed due to widespread hypertension, can modulate bone metabolism through effects on osteoblast and osteoclast activity, immune regulation, and neurohormonal pathways [5]. Agents such as beta-blockers, thiazide diuretics, angiotensin-converting enzyme inhibitors (ACEIs), angiotensin II receptor blockers (ARBs), and calcium channel blockers (CCBs) may exert protective effects on bone density and remodeling [6].

Hypercholesterolemia, a prevalent metabolic disorder, is frequently managed with antihyperlipidemic agents, particularly statins [7]. Beyond lipid-lowering, statins possess pleiotropic properties such as anti-inflammatory, immunomodulatory, and osteoanabolic effects [7]. Experimental and clinical studies have shown that statins enhance osteoblast activity, inhibit osteoclastic resorption, promote angiogenesis, and improve bone-implant contact, supporting osseointegration and implant stability [8]. These findings indicate a potential protective role of statins in implant therapy among patients with dyslipidemia [9].

Mixed antidepressant medications, particularly selective serotonin reuptake inhibitors (SSRIs), are increasingly prescribed for mood and anxiety disorders [10]. These medications have been linked to adverse skeletal effects, including increased bone resorption, reduced bone formation, decreased bone mineral density, and higher fracture risk [11]. Emerging evidence suggests that mixed antidepressant therapy may impair peri-implant bone healing and elevate implant failure rates, particularly during early osseointegration [11].

Given the widespread and often chronic use of antihypertensive, mixed antidepressants, and antihyperlipidemic agents, understanding their effects on dental implant outcomes is of high clinical relevance. Therefore, the aim of this study is to evaluate the impact of these commonly prescribed medications on the long-term failure and survival rates of dental implants.

## Materials and methods

Patients and data sources

This study was carried out in compliance with the Strengthening the Reporting of Observational Studies in Epidemiology (STROBE) guidelines [12] and in accordance with the ethical principles of the Declaration of Helsinki (1975, revised 2013) [13]. The study protocol was approved by the Ethics Committee of Saint Joseph University of Beirut (approval number: CER-2024-245). All patients provided informed consent allowing a retrospective analysis of medical records and digital radiographic archives of dental implants.

A database was created using Microsoft Excel® software (Microsoft Corp., Redmond, WA, USA) to record the identification numbers of patients who received dental implants between January 1, 2012, and December 31, 2022. Patient records were subsequently retrieved from clinical archives for manual review. Data collected included implant characteristics (diameter and length) and surgical details, such as guided bone regeneration and sinus membrane elevation procedures.

Preoperative information, including general and oral health status, medical history (type, dosage, and duration of medication use), sociodemographic characteristics, parafunctional habits, and behavioral factors, was obtained through a standardized self-administered questionnaire completed during the initial consultation. Intraoperative and postoperative periapical radiographs were also reviewed. Patients were then selected according to predefined inclusion and exclusion criteria.

Inclusion criteria

Patients classified according to the American Society of Anesthesiologists (ASA) physical status I or II [14], without systemic conditions or metabolic bone disease other than hypertension, dyslipidemia, or depression, and who were non-smokers and non-alcoholics, were included. Participants had stable periodontal health, good oral hygiene (bleeding on probing (BOP) < 10%), and no occlusal parafunction. Only patients with follow-up periapical radiographs and implants measuring 3.5 mm in diameter were included. Individuals who were not taking multiple medications, not pregnant, and receiving only a single drug therapy, specifically antihypertensive, mixed antidepressant, or antihyperlipidemic agents, were considered eligible for inclusion in the study.

Exclusion criteria

Patients with severe systemic diseases (ASA III or IV) [14], Paget’s disease, uncontrolled hyperthyroidism, diabetes, hypertension, dyslipidemia, depression, cancer, corticosteroid therapy, antiepileptic drugs, or vitamin D deficiency were excluded. Additional exclusions included stage 3 or 4 periodontal disease, pathological occlusal parafunctions, pregnancy, implants < 3.5 mm in diameter in posterior regions, inadequate radiographs, poor oral hygiene (BOP > 10%), non-cooperation, non-conical implants, rough surfaces not treated by sandblasting and double etching, implants not approved by the the US Food and Drug Administration (FDA), and patients consuming alcohol or tobacco.

Study population

**Figure 1:**
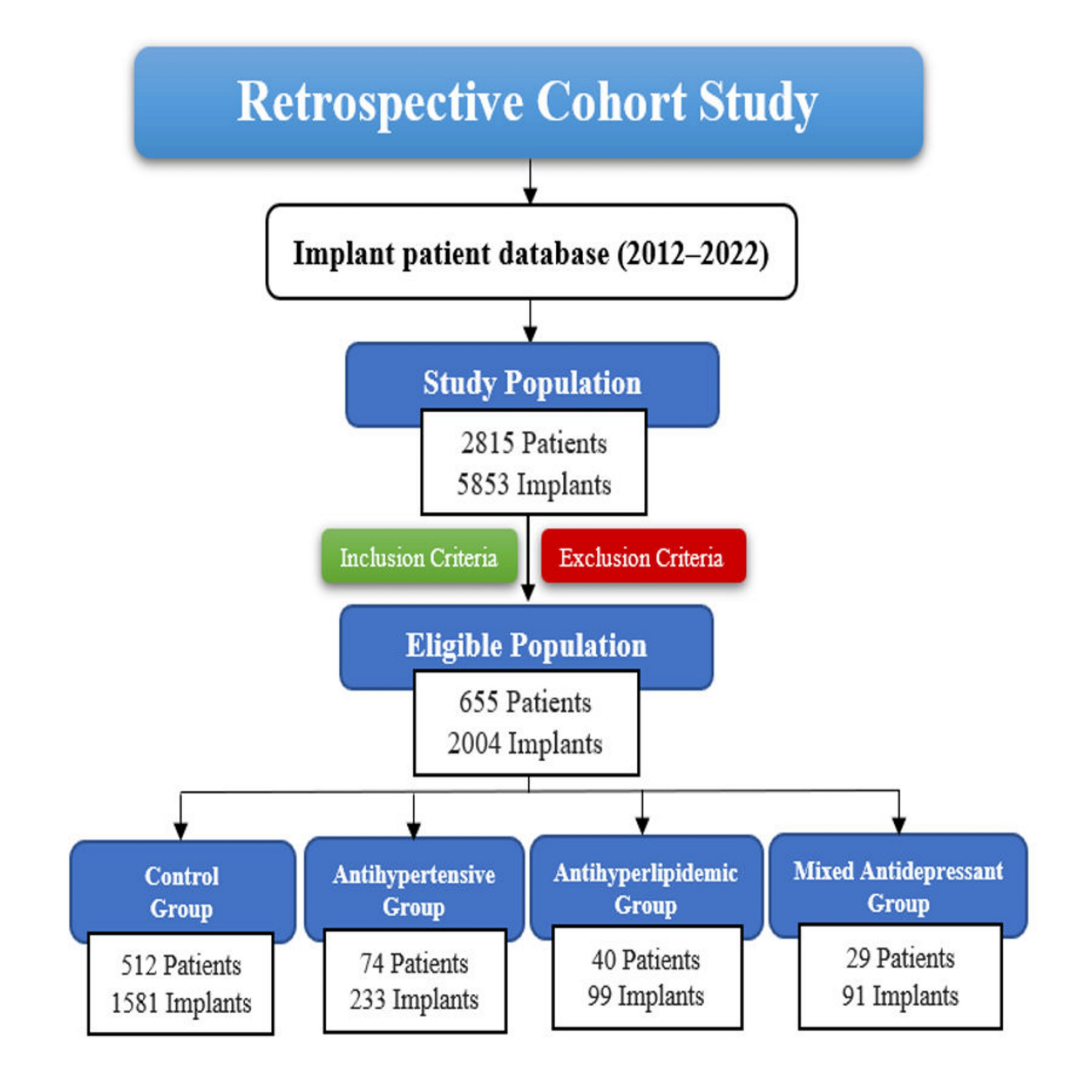
Flow diagram of the participant selection process

Of the 2,815 patients who received a total of 5,853 implants during the study period, 655 patients with 2,004 implants met the eligibility criteria and were assigned to four groups. The control group included non-medicated patients (512 patients; 1,581 implants). The antihypertensive group comprised patients receiving only antihypertensive therapy, including thiazide diuretics, beta-blockers, CCBs, ACEIs, and ARBs (74 patients; 233 implants). Group 2 (antihyperlipidemic group) consisted of patients receiving only lipid-lowering therapy, primarily statins (40 patients; 99 implants). Group 3 (mixed antidepressant group) included patients receiving only mixed antidepressants, such as selective serotonin reuptake inhibitors (SSRIs) (29 patients; 91 implants) (Figure 1). 

Implant placement and postoperative protocols

Implant surgery was performed under local anesthesia (1.8 ml of 4% Articaine with 1:100,000 epinephrine). For patients with insufficient bone volume, bone augmentation procedures (e.g., lateral bone grafting, sinus membrane elevation) were performed six months prior to implant placement.

Postoperatively, patients were advised to rinse three times daily for 7 days with 0.12% chlorhexidine (CHX) solution and follow a soft diet. A prophylactic antibiotic (1 g Amoxicillin®, BID) for 7 days was prescribed. Analgesics (Paracetamol®, 2 × 500 mg every 6 hours as needed) were provided. Follow-up examinations were conducted 7-10 days post-surgery for suture removal and reinforcement of oral hygiene instructions. Peri-implant bone levels were assessed radiographically and clinically by monitoring bone resorption and implant mobility.

Study outcomes and follow-up

The primary objective of this study was to evaluate implant failure rates across the different groups and to assess implant survival at long-term follow-up. Patients were followed until implant failure, death, or loss to follow-up prior to the end of the study period. Implant failure was defined according to the Pisa Consensus Conference guidelines of the International Congress of Oral Implantologists (ICOI). Implants were classified as failed if they exhibited functional pain, mobility, radiographic bone loss of ≥50% of the implant length, uncontrolled exudate, or were absent from the oral cavity.

Follow-up duration was calculated from implant placement to the most recent clinical examination. Clinical and radiographic evaluations monitored implant stability, peri-implant bone loss, signs of infection, and peri-implant bone levels. Radiographic assessment was performed by a single operator using periapical radiographs obtained with the long-cone paralleling technique and Rinn film holders (0.25 s, 65 kV, 2 mA). Medication usage was validated at each recall. Plaque control, periodontal status, frequency and quality of supportive peri-implant care (SPIC), and restoration cleanability were also assessed. Radiographs were archived digitally using Soft-Dent DBSWIN® (Dürr Dental, Bietigheim-Bissingen, Germany), with measurements calibrated to implant length to ensure accurate evaluation of the distance between the bone crest and implant head. Implant failures and their causes were collected from patient implant records.

Statistical analysis

Statistical analyses were performed using IBM SPSS Statistics for Windows, Version 25.0 (Released 2017; IBM Corp., Armonk, NY, USA). Quantitative variables were expressed as means, standard deviations (SDs), and 95% confidence intervals (CIs), while qualitative variables were reported as frequencies and percentages. Statistical testing was conducted in three stages. First, implant survival was assessed using Kaplan-Meier survival curves, and differences between the monotherapy groups (antihypertensive, antihyperlipidemic, and mixed antidepressant groups) and the control group were evaluated using the log-rank test. Second, implant failure rates were analyzed using a fixed-effects logistic regression model and a generalized linear mixed model (GLMM) to examine the time interval between implant placement and failure. Finally, an Omnibus test was applied to evaluate the influence of potential confounding variables, including oral hygiene and deleterious habits, on the study outcomes. A p-value below the conventional threshold of 0.05 indicates that the difference between groups is statistically significant, allowing rejection of the null hypothesis.

## Results

Kaplan-Meier survival curve analysis

In the present study, Kaplan-Meier survival curves illustrate the cumulative survival probability of dental implants over time for the control group (blue curve) compared with each of the three monotherapy groups (red curve). The y-axis represents implant survival probability (ranging from 0 to 1), and the x-axis represents time in years. All curves begin at a survival probability of 1 (100%) at baseline and demonstrate changes in implant survival over a 10-year observation period.

To ensure a balanced and statistically robust comparison, survival analyses were restricted to a 10-year follow-up period. This approach minimized potential bias arising from unequal or extended follow-up durations across groups and provided a stable comparison between the control and monotherapy groups. Consequently, it allowed a clearer evaluation of the potential beneficial or detrimental effects of antihypertensive agents, antihyperlipidemic drugs, and mixed antidepressants on long-term implant survival.

**Figure 2:**
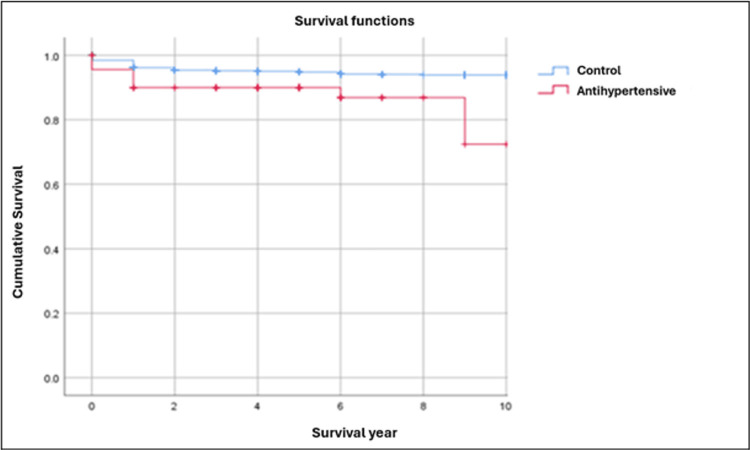
Kaplan-Meier survival curves comparing dental implant survival rates between the control group and the antihypertensive group

**Figure 3:**
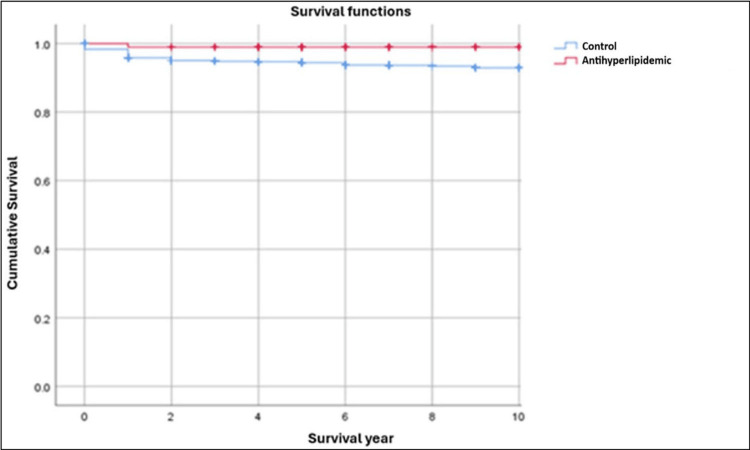
Kaplan-Meier survival curves comparing dental implant survival rates between the control group and the antihyperlipidemic group

**Figure 4:**
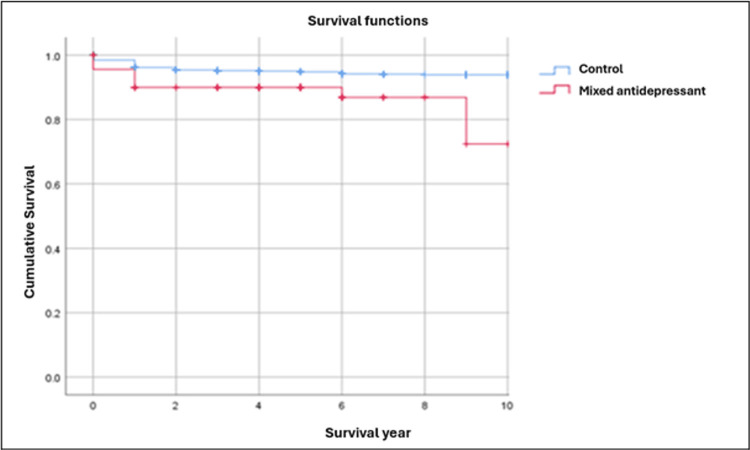
Kaplan-Meier survival curves comparing dental implant survival rates between the control group and the mixed antidepressant group

Visual inspection of the Kaplan-Meier curves demonstrated a progressive decline in implant survival probability across all groups. Patients receiving antihypertensive therapy exhibited higher implant survival than the control group (Figure 2). Similarly, patients receiving antihyperlipidemic therapy maintained consistently higher survival throughout the observation period compared with the control group (Figure 3). In contrast, patients receiving mixed antidepressant therapy had lower implant survival than the control group (Figure 4).

Survival analysis: log-rank test

The log-rank test was used to compare implant survival distributions between the control group and the monotherapy groups (antihypertensive, antihyperlipidemic, and mixed antidepressant groups). For the antihypertensive group, the chi-square value was 8.406 with a p-value of 0.004, indicating a significant difference compared with the control group. For the antihyperlipidemic group, the chi-square value was 3.966 with a p-value of 0.046, indicating a significant difference. Finally, for the mixed antidepressant group, the chi-square value was 8.467 with a p-value of 0.004, confirming a significant difference relative to the control group (Table 1).

**Table 1:** Overall comparison of implant survival distributions between each monotherapy group and the control group using the log-rank (Mantel-Cox) test *Reference value: control group

Global comparisons	Log rank (Mantel-Cox)		
Testing the equality of implant survival distributions for the different groups of:	Chi-square	df	Sig.
Antihypertensive	8.406	1	0.004*
Antihyperlipidemic	3.966	1	0.046*
Mixed antidepressant	8.467	1	0.004*

Fixed-effects logistic regression model

The fixed-effects logistic regression model for each monotherapy group demonstrated a statistically significant difference compared with the control group (Table 2).

**Table 2:** Fixed-effects logistic regression model for each monotherapy group

Model term	Coefficient	Standard error	t	Sig.	95% CI	
					Lower limit	Upper limit
Intercept	-2.916	0.1141	-25.562	0	-3.14	-2.692
Antihypertensive	-1.392	0.5923	-2.351	0.019	-2.554	-0.231
Antihyperlipidemic	-1.638	1.0117	-1.619	0.011	-3.622	0.346
Mixed antidepressant	0.932	0.3412	2.731	0.006	0.263	1.601

Table 3 presents the results of a fixed-effects logistic regression model used to predict the probability of implant failure for each group. In the control group, the failure rate was 5.12% (n = 81/1,581). The antihypertensive group showed a failure rate of 1.29% (n = 3/233), the antihyperlipidemic group had a failure rate of 1.01% (n = 1/99), and the mixed antidepressant group exhibited a failure rate of 12.09% (n = 11/91). Using the control group as the reference, logistic regression indicated reduced odds of implant failure in the antihypertensive (OR = 0.248) and antihyperlipidemic groups (OR = 0.194), whereas the mixed antidepressant group exhibited increased odds of implant failure (OR = 2.54), reflecting a higher risk relative to the control (Table 3).

**Table 3:** Logistic regression model of implant failures for each group

Model term	Failure (yes)	Exp. (coefficient)	95% CI	
	n (%)		Lower limit	Upper limit
Control	81 (5.12)	0.054	0.043	0.068
Antihypertensive	3 (1.29)	0.248	0.078	0.794
Antihyperlipidemic	1 (1.01)	0.194	0.027	1.414
Mixed antidepressant	11 (12.09)	2.54	1.301	4.958

Analysis of the GLMM

**Figure 5:**
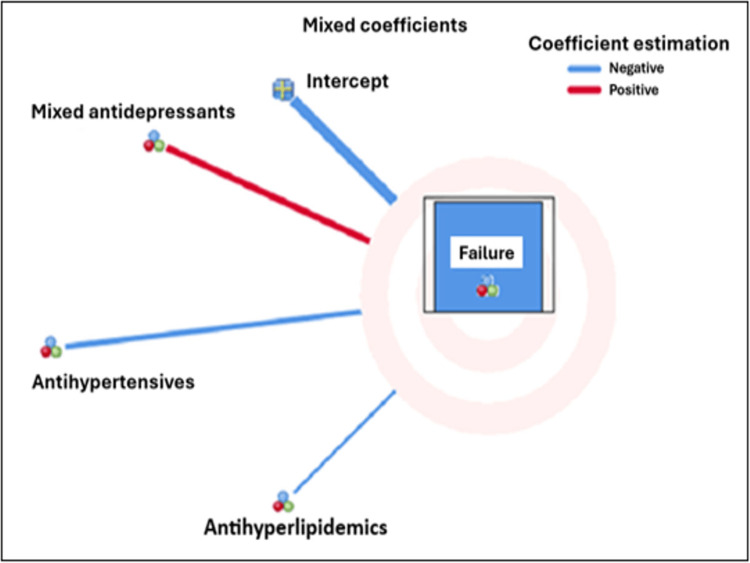
GLMM analysis of fixed-effect coefficients for implant failures in each single-drug group This image was generated using IBM SPSS Statistics for Windows, Version 26.0 (Released 2018; IBM Corp., Armonk, NY, USA), with advanced data processing executed via the integrated Python 3.4 distribution (Python Software Foundation, Fredericksburg, VA, USA).

The GLMM was used to compare the control group, which served as the reference, with the three monotherapy groups. With respect to implant failure, the mixed antidepressant group showed a positive coefficient (red), indicating an increased risk of implant failure, whereas the antihypertensive and antihyperlipidemic groups showed negative coefficients (blue), indicating a reduced risk of implant failure (Figure 5).

Omnibus tests of model coefficients

The Omnibus test was conducted to assess the overall significance of the model and facilitate data interpretation. Risk analysis demonstrated an association between the four study groups and dental implant survival. A parametric multilevel mixed-effects survival model was applied to adjust for potential confounding variables, including oral hygiene and detrimental habits; none of these covariates showed a statistically significant effect on the impact of the antihypertensive, antihyperlipidemic, or mixed antidepressant groups on implant survival.

With respect to medication, patients in the mixed antidepressant group exhibited a significantly higher risk of implant failure compared with the control group (HR = 2.17; 95% CI: 1.17-4.04). In contrast, the antihyperlipidemic group was associated with a reduced risk of implant failure (HR = 0.16; 95% CI: 0.02-1.15), as was the antihypertensive group (HR = 0.23; 95% CI: 0.07-0.74) (Table 4).

**Table 4:** Omnibus tests: Cox regression model for implant failure factors *p-value of the Omnibus test < 0.05.

Variable	Risk ratios	Risk ratio confidence at 95%	p-value
Antihypertensive	0.23	0.07-0.74	0.013*
Antihyperlipidemic	0.16	0.02-1.15	0.048*
Mixed antidepressant	2.17	1.17-4.04	0.014*

The Omnibus test p-value (<0.05) indicates that the overall model is statistically significant, confirming the association between the studied groups, implant failure, and long-term implant survival (Table 4).

## Discussion

Dental implants are considered a first-line therapy for the replacement of missing teeth, with reported long-term survival rates ranging from 90% to 95% [3]. As implant therapy becomes increasingly common, more patients with systemic medical conditions are undergoing treatment [15]. Systemic diseases and the medications used to manage them may influence osseointegration and implant survival. Although previous studies have highlighted the impact of chronic systemic disorders and long-term medication use on implant outcomes [15], our study is among the first to suggest that certain systemic medications may enhance long-term implant survival. These findings may have implications for future pharmacological strategies aimed at optimizing implant success and potentially influencing implant design and biomaterial development.

Antihypertensives 

Hypertension is a chronic condition commonly treated with beta-blockers, thiazide diuretics, ACEIs, and ARBs [16]. These agents are widely prescribed, particularly within the Lebanese population, and have been shown to positively influence bone metabolism and healing [5].

In our cohort, the antihypertensive group demonstrated significantly higher long-term implant survival compared with the control group. Implant failure rates were 1.29% (n = 3/233) in the antihypertensive group versus 5.12% (n = 81/1,581) in the control group (Table 3). Kaplan-Meier analysis showed consistently higher cumulative survival in the antihypertensive group (Figure 2), and the log-rank test confirmed a statistically significant difference (chi-square = 8.406; p = 0.004) (Table 1). GLMM analysis and Cox regression further supported this protective association, with a hazard ratio of 0.23 (95% CI: 0.07-0.74) (Table 4).

These findings align with previous clinical and experimental research. Manor et al. [17] and Wu et al. [5] reported reduced implant failure rates among patients receiving antihypertensives. Experimental studies, including those by Al Subaie et al. [18] and Sadr et al. [19], demonstrated enhanced bone remodeling, increased osteoblast activity, and reduced osteoclast function. Additional retrospective and review studies have similarly confirmed improved implant outcomes in patients taking renin-angiotensin system inhibitors and beta-blockers [4,20-22]. Collectively, the evidence suggests a protective role of antihypertensives in implant osseointegration and long-term survival.

Antihyperlipidemics

Statins, lipid-lowering agents that inhibit HMG-CoA reductase [23], have demonstrated pleiotropic effects on bone metabolism. In the present study, the use of antihyperlipidemic agents was associated with improved implant survival. Failure rates were 1.01% (n = 1/99) among statin users compared with 5.12% (n = 81/1581) in controls (Table 3). Kaplan-Meier curves showed consistently higher survival probabilities for statin users (Figure 3), and the log-rank test confirmed statistical significance (chi-square = 3.966; p = 0.046 < 0.05) (Table 1).

Multilevel survival modelling further demonstrated a reduced risk of implant failure in the statin group (HR = 0.16; 95% CI: 0.02-1.15) (Table 4). Although the confidence interval suggests some variability, the overall trend indicates a protective association.

These findings are consistent with prior studies and meta-analyses reporting enhanced bone mineral density, improved bone-implant contact, and stimulated osteogenesis following systemic or local statin administration [8,9,24,25]. Together, these data support the hypothesis that antihyperlipidemic agents may positively influence osseointegration and implant longevity.

Mixed antidepressants

Depression is a prevalent psychiatric disorder frequently treated with mixed antidepressants, including SSRIs. These medications have been associated with reduced bone mineral density and increased fracture risk [26]. Serotonin receptors are present in peripheral tissues, including osteoblasts [11], and serotonin reuptake inhibition has been shown to increase osteoclast differentiation while reducing osteoblast proliferation, thereby impairing bone formation [11].

Our findings demonstrate a negative association between mixed antidepressant use and implant survival. Implant failure occurred in 12.09% (n=11/91) of patients in the mixed antidepressant group compared with 5.12% (n=81/1581) in controls (Table 3). Kaplan-Meier analysis showed consistently lower cumulative survival in this group (Figure 4), and the Log-Rank test confirmed strong statistical significance (Chi-square = 8.467; p = 0.004) (Table 1). Cox regression analysis further demonstrated a significantly increased risk of implant failure (HR = 2.17; 95% CI: 1.17-4.04) (Table 4).

These results are consistent with existing literature. Abu Nada et al. [10] demonstrated impaired osseointegration in animal models receiving SSRIs. Altay et al. [27] reported a threefold increase in implant failure among SSRI users, while Silva et al. [28] and Shariff et al. [29] confirmed significantly elevated failure risks in systematic reviews and meta-analyses. The cumulative evidence indicates that mixed antidepressants may adversely affect bone metabolism and implant stability, emphasizing the importance of thorough medical evaluation and patient counselling during implant planning [27,29].

Strengths and limitations

Our study is the first to compare three single-drug groups (antihypertensive, antihyperlipidemic, and mixed antidepressant) with healthy controls in relation to long-term implant survival. By focusing on mono-medicated groups, the study design reduced potential confounding from pharmacological interactions commonly observed in previous retrospective cohorts.

Comprehensive statistical modelling, including Kaplan-Meier analysis, GLMM, Cox regression, and Omnibus testing, strengthened the robustness of the findings. Multilevel survival modelling allowed adjustment for potential confounders, and oral hygiene did not demonstrate a statistically significant effect on implant survival within the studied groups.

Additionally, adherence to antihypertensive, antihyperlipidemic, and mixed antidepressant regimens is generally high (up to 88%), and their skeletal effects are primarily time-dependent rather than strictly dose-dependent, thereby reinforcing the clinical validity of our results [30].

Nevertheless, several limitations should be acknowledged. The retrospective design and reliance on self-reported medical information may have introduced recall bias. Additionally, group imbalance, together with the absence of randomization and blinding, may have contributed to selection bias.

## Conclusions

Dental implants have achieved remarkable success over the past three decades, becoming a preferred solution for dental rehabilitation. Optimal bone remodeling and proper healing during the early phases of osseointegration are crucial for long-term implant longevity and functionality. Multiple factors influence bone healing and osseointegration and may potentially lead to implant failure, including chronic medication use. Our statistical analyses indicated that mixed antidepressants are associated with an increased risk of implant failure, whereas antihypertensive and antihyperlipidemic agents appear beneficial, promoting better implant survival, likely through protective effects on bone vascularization and metabolism.

Although some studies demonstrate direct drug effects on osseointegration, most current research is based on in vitro experiments, animal models, and retrospective studies. Randomized prospective clinical trials are necessary to establish evidence-based guidelines regarding the impact of chronic medications, particularly antihypertensive, antihyperlipidemic, and mixed antidepressant agents, on osseointegration and long-term implant survival. Such research could help clinicians better understand the impact of these medications on implant success, thereby leading to more precise guidelines and improving the management of dental implant patients while minimizing medication-related risks.
